# A meta-analysis of declines in local species richness from human disturbances

**DOI:** 10.1002/ece3.909

**Published:** 2013-12-12

**Authors:** Grace E P Murphy, Tamara N Romanuk

**Affiliations:** Department of Biology, Dalhousie UniversityHalifax, Nova Scotia, Canada

**Keywords:** Biodiversity loss, disturbance, extinction, habitat loss, species invasions, species richness

## Abstract

There is high uncertainty surrounding the magnitude of current and future biodiversity loss that is occurring due to human disturbances. Here, we present a global meta-analysis of experimental and observational studies that report 327 measures of change in species richness between disturbed and undisturbed habitats across both terrestrial and aquatic biomes. On average, human-mediated disturbances lead to an 18.3% decline in species richness. Declines in species richness were highest for endotherms (33.2%), followed by producers (25.1%), and ectotherms (10.5%). Land-use change and species invasions had the largest impact on species richness resulting in a 24.8% and 23.7% decline, respectively, followed by habitat loss (14%), nutrient addition (8.2%), and increases in temperature (3.6%). Across all disturbances, declines in species richness were greater for terrestrial biomes (22.4%) than aquatic biomes (5.9%). In the tropics, habitat loss and land-use change had the largest impact on species richness, whereas in the boreal forest and Northern temperate forests, species invasions had the largest impact on species richness. Along with revealing trends in changes in species richness for different disturbances, biomes, and taxa, our results also identify critical knowledge gaps for predicting the effects of human disturbance on Earth's biomes.

## Introduction

Developing the ability to predict the consequences of environmental change is one of the most significant challenges in ecology today (Chapin et al. 1997; Pereira et al. 2010; Dawson et al. 2011). Evidence is increasingly demonstrating the negative effects of biodiversity loss on Earth's ecosystem processes (Loreau et al. 2001; Balvanera et al. 2006; Wardle et al. 2011; Hooper et al. 2012). Given the increasing human domination of Earth's biomes, establishing accurate estimates of the magnitude of biodiversity loss resulting from common human disturbances, such as land-use change and habitat loss, species invasions, climate change, and nutrient additions, is of particular importance.

With the sustainability of human life on Earth relying on the services that healthy ecosystems provide (Millenium Ecosystem Assessment 2005), a better understanding of why and how species are being lost from ecosystems is needed. There is considerable uncertainly however over the magnitude of current and future biodiversity loss (Barnosky et al. 2012). Previous attempts to estimate changes in biodiversity have relied heavily on expert opinion (Sala et al. 2000) or have focused on estimating extinction risks for particular taxa (Thuiller et al. 2005). Potential time lags between environmental change and extinctions (Krauss et al. 2010), differences in extinction rate estimates based on species-area-curves (He and Hubbell 2011), and other confounding effects have made predicting the magnitude of species loss resulting from various human-caused disturbances problematic (Bellard et al. 2012). Differences between modeling approaches and uncertainties within model projections have also resulted in widely varying predictions of future biodiversity change (Pereira et al. 2010). For example, two modeling approaches used to project the future global extinction risks for birds revealed very different estimates with Jetz et al. (2007) projecting 253–455 species at risk for extinction by the year 2100 while Sekercioglu et al. (2008) projects 2150 species at risk for extinction in the same time period.

One potential solution for the uncertainties in estimating biodiversity loss is making use of studies that report the difference in species richness between disturbed and undisturbed habitats. Species richness is not synonymous with biodiversity, with the later serving as a more complex description of both the variation in the number of species and their relative abundances, along with genetic and ecosystem variation. However, declines in species richness can be an indicator of biodiversity loss and with studies that examine changes in species richness following disturbances among the most common in the ecological literature, compiling these studies and analyzing changes in species richness can provide information on the potential biodiversity loss occurring from human-caused disturbances. In this study, we have compiled studies that document the effects of human-caused disturbances on changes in species richness into a dataset that includes 327 empirical values of change in species richness taken from 245 previously published experimental and observational disturbance studies. Using a combination of categorical and continuous meta-analyses, we determined whether there are differences in the fraction of change in species richness resulting from five anthropogenic disturbances: species invasions, nutrient addition, temperature increase, habitat loss or fragmentation, and land-use change. We also determined whether the fraction of change in species richness caused by the disturbances differed based on: (1) the type of biome (Northern temperate forest, boreal forest, tropical, or aquatic); (2) the type of species (producer, ectotherm, or endotherm); (3) the type of study (experimental or observational); (4) the initial species richness; (5) the latitude of the study site; and (6) the length of the experiment.

## Methods

### Selection criteria

**Figure 1:**
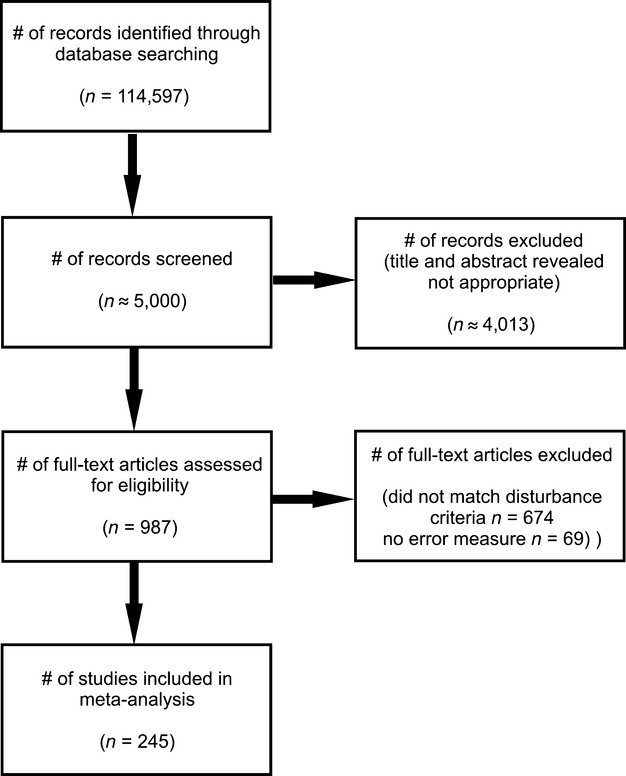
Preferred Reporting Items for Systematic reviews and Meta-analyses (PRISMA) diagram. PRISMA flow diagram showing an overview of the study selection process.

Our dataset was compiled by searching the biological literature for studies that reported the effects of anthropogenic disturbances on species richness. We focused on five anthropogenic disturbances that have been identified as major drivers of current biodiversity decline: species invasions, nutrient addition, temperature increase, habitat loss or fragmentation, and land-use change (Vitousek et al. 1997; Jackson et al. 2001). We performed a literature search using the ISI Web of Science database of the following research areas: “environmental sciences ecology”, “biodiversity conservation”, and “marine freshwater biology”. We used the following search expressions: “biodiversity loss” OR “species loss” OR “species richness” OR “community change” AND (“invasi* species” OR “habitat loss” OR “land use change” OR “climate change” OR “experiment* warm*” OR increas* temperature” OR “eutrophication” OR “nutrient add*”). A final search of the literature was completed on 10 February 2013. We searched for studies that experimentally manipulated disturbances (*n* = 113) or observational studies that compared a disturbed habitat with a control (undisturbed) habitat (*n* = 214). The literature search yielded 114,597 citations, of which 245 studies that included 327 values of change in species richness were included in the final dataset (Fig. 1). All papers reported a mean measure of species richness and a corresponding error measure in both a disturbance and a reference condition. Values were given in 147 of the responses. For studies that did not explicitly state results but instead showed results in a figure, as was the case for 180 responses, the average species richness and corresponding error measures were estimated using GetData Graph Digitizer software (S. Fedorov, Russia). If a study presented multiple responses, these were only included when the responses were for different disturbance categories, different geographical regions, or different trophic categories. Multiple responses that did not differ from each other based on these criteria were averaged, and the average response was used in the dataset. We also averaged responses for studies that manipulated disturbance over a range of disturbance intensities. Because we had no way of separating the effects of multiple disturbances, we only included responses that gave the effects of single disturbances. If a combined disturbance effect was given, the response was not included in the dataset. We took data from the final sampling date for studies that measured species richness over a period of time.

We followed strict guidelines in choosing the types of disturbance studies to be included in the analysis. For the temperature increase category, we only included studies that increased temperature per se (e.g., Chapin et al. 1995). Studies that combined other climate change effects, such as altered light and precipitation, with increases in temperature were not included (e.g., Zhou et al. 2006) nor were observational studies which compared natural communities growing in areas that differ in ambient temperature (e.g., Kennedy 1996). For nutrient addition, we included studies that enriched the experimental community with nitrogen (e.g., Bonanomi et al. 2009), phosphorus (e.g., Cherwin et al. 2008), or a fertilizer solution containing one or both of these nutrients (e.g., Lindberg and Persson 2004). Habitat loss and land-use change comprised two separate categories, each with their own subcategories. We classified a disturbance as a form of habitat loss if the habitat had been fragmented or reduced in size. If the habitat size remained the same but was transformed from a natural habitat to either an urban or agricultural habitat the disturbance was classified as land-use change. For habitat loss, we included studies that fragmented experimental plots (e.g., Gonzalez and Chaneton 2002), those where habitat size had been reduced (Bonin et al. 2011), or those that compared communities present in control sites to those that had been clear cut or logged (e.g., Biswas and Malik 2010). We did not include studies that combined corridor effects with fragmentation (e.g., Rantalainen et al. 2004). We grouped three habitat loss categories (fragmentation, reduction in habitat size, and logging) into a single habitat loss category. While the fraction of change in species richness did differ between the three categories (fragmentation = 13% decline, *n* = 21; reduction in habitat size = 25% decline, *n* = 22; logging = 30% decline, *n* = 15), the difference was not statistically significant, likely due to the high variability within categories caused by low sample sizes (*Q*_b_ = 1.96, *P* = 0.38). We decided to group together these three habitat categories to increase the overall sample size for the habitat loss category. All studies in the land-use change category were studies that observed species richness in a site that had been transformed from a natural area to one dominated by human development (e.g., urban or suburban areas) or agricultural activity, compared with a reference natural area. The fraction of change in species richness differed between the two land-use change categories (human development = 19% decline, *n* = 21; agricultural activity = 48% decline, *n* = 39); however, the difference was not statistically significant (*Q*_b_ = 3.05, *P* = 0.081); thus, we grouped the two types into a single land-use change category to increase the sample size for this category. Finally, for species invasions, we included studies in which a non-native species, or group of non-native species, was added (intentionally or unintentionally) to an established community. We did not include studies that examined the effects of removing non-native species from previously invaded communities (e.g., Ostertag et al. 2009). We also included observational studies that examined an uninvaded site with an invaded site. We only included native species richness for the invasion studies.

We grouped studies into one of three species categories. Producers included both terrestrial and aquatic primary producers, ectotherms included animals that rely on external sources to control body temperature, and endotherms included animals that produce heat internally. We chose these three species categories as we wanted to be more specific than simply grouping species as consumers or producers yet separating the studies into anything more specific than these three categories would have resulted in very small sample sizes for each category. Categorizing the consumer species as ectotherms and endotherms takes into account differences in metabolic activity and body size, as endotherms are generally larger bodied animals compared with ectotherms.

The 245 studies spanned most of the Earth's biomes. Ten terrestrial biomes were classified into condensed ecoregions (Bailey 1998): arctic, alpine, northern temperate forest, southern temperate forest, boreal forest, savanna, mediterranean, desert, grassland, and tropical. Freshwater, marine, estuary, and wetland ecosystems were combined into an aquatic biome category. In the categorical analysis of the biomes, effects were only calculated for disturbance-biome combinations that included five or more responses. Thus, effect sizes were not calculated for 36 of the 55 disturbance-biome combinations, as they did not fit this minimum sample size. In order to make relevant comparisons across biomes, we only analyzed biomes that contained effect sizes for at least three of the five disturbances. This left four biomes in the analysis: northern temperate forest, boreal forest, tropical, and aquatic. The study site latitude was also recorded for each response to examine any potential latitudinal gradients in species loss.

### Data analysis

We performed weighted random effects meta-analyses using MetaWin 2.0 software (Rosenberg and Adams 2000). We considered a random effects analysis, which assumes that effect sizes will exhibit random variation among studies, to be more appropriate than a fixed effects analysis as the studies included in our dataset vary widely in both methodology and biological factors. We used the standard equation for the response ratio (RR) as the effect size for the analyses to compare species richness (SR) between experimental (*e*) and control (*c)* conditions. The response ratio is calculated as:


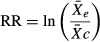


The response ratio is a common effect size measure in ecological meta-analyses (Hedges et al. 1999). Response ratios that are significantly greater or less than zero indicate a larger change in species richness between the control and disturbance treatments, with the direction of change indicating whether the disturbance increased or decreased species richness relative to the reference condition. The percentage of change in the responses that we refer to in the text was calculated as:


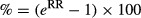


The independent responses in the analyses were weighted according to their sample variances to account for the difference in statistical precision between individual experiments (Hedges et al. 1999). Greater weight is given to experiments whose estimates have a smaller standard error, thus a greater precision. Variance for each response was calculated as:


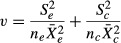


We used a combination of categorical and continuous meta-analyses to test for the effect of seven different factors on the magnitude of change in species richness between the control and disturbance treatments. The factors were as follows:

1. Disturbance type (categorical). This factor included five disturbance type categories: habitat loss, land-use change, species invasion, nutrient addition, and temperature increase.
2. Study type (categorical). This factor included two study type categories: experimental and observational, but only compared between habitat loss and species invasions, as these were the only two disturbances to contain response from both study types.
3. Species category (categorical): This factor included three species categories: producers, ectotherms, and endotherms.
4. Biome type (categorical): This factor included four biome categories: northern temperate forest, boreal forest, tropical, and aquatic.
5. Initial species richness (continuous): Initial species richness was given as the species richness in the control treatment for each response.
6. Latitude (continuous): Latitude of the study site was given for each response.
7. Experimental length (continuous): Length (in days) was given for each of the experimental responses. Observational studies were not included in this analysis.

We used 95% confidence intervals to determine significant differences in an effect size from zero, indicating an increase or decrease in species richness in the disturbed treatment compared with the control. If the confidence interval overlaps with zero then the species richness did not significantly increase or decrease in that response. We also used 95% confidence intervals to compare between the different categories within a factor. If the intervals of two categories overlapped then they are said to not significantly differ in their magnitude of species richness change. In categorical meta-analysis, one can test whether the effect sizes of the categories within a factor are homogeneous, meaning that the observed differences are due to sampling error and not due to the effect of the category by examining the heterogeneity statistic (*Q*). The total heterogeneity for a group of comparisons (*Q*_t_) is partitioned into within-group heterogeneity (*Q*_w_) and between-group heterogeneity (*Q*_b_). A significant between-group heterogeneity statistic indicates that the effect sizes between the different categories in a factor are significantly heterogeneous, and thus, the differences are not due to sampling error alone. In the continuous meta-analysis models, we used the model heterogeneity (*Q*_m_) to determine whether the relationship between the magnitude of species loss and the continuous variable was significant. A significant *Q*_m_ indicates that the model explains a significant amount of variability within effect sizes.

### Publication bias

Publication bias occurs when there is a tendency toward publishing only significant results, leading to a disparity in the strength or direction of the results of published studies compared with those of unpublished studies (Moller and Jennions 2001). We used two methods to test for publication bias in our dataset. The first was visual inspection of a “funnel plot” of sample size against effect size. If the effect sizes were derived from a random sample of studies, suggesting that publication bias is low, the plot should reveal a funnel shape, with small sample sizes showing a larger variance in individual effects and a decrease in variance with increasing sample size (Moller and Jennions 2001). The second method we used to test for publication bias was the calculation of a fail-safe number (Rosenthal 1991). The fail-safe number provides an estimate of the number of future studies needed to change a significant effect to a non-significant one (Moller and Jennions 2001). Therefore, a larger fail-safe number relates to a lower chance of publication bias. Rosenthal (1991) has suggested that a fail-safe number that is equal to or greater than 5*n* + 10 (where *n* is the number of studies) provides evidence of a robust effect size that is not skewed by publication bias.

## Results

**Figure 2:**
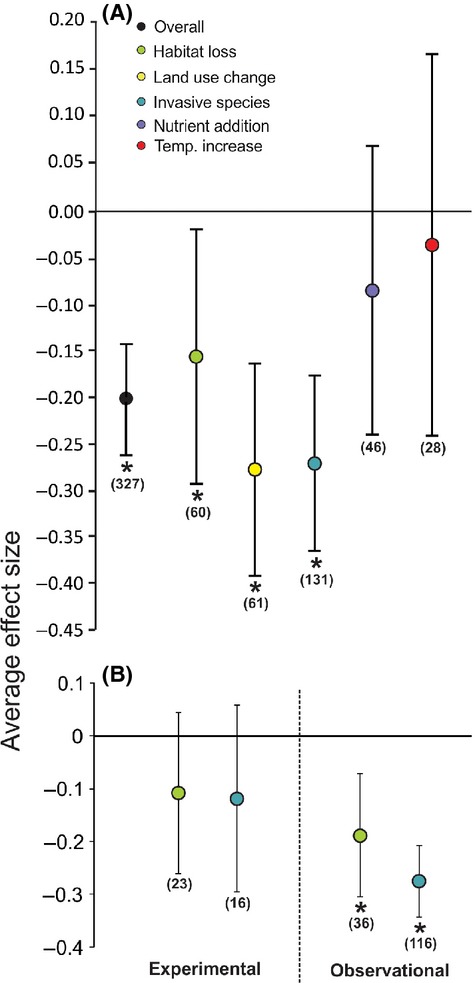
Change in species richness following anthropogenic disturbances. (A) Average response ratios and 95% confidence intervals of species richness across all disturbance types and for each individual disturbance. (B) Average response ratios and 95% confidence intervals of species richness between experimental and observational studies for habitat loss and species invasions across all biomes. The values in parentheses represent the number of responses included in the analysis. Values that significantly differ from zero, according to the 95% confidence intervals, are indicated with an asterisk.

Our results show that, on average, human disturbances lead to an 18.3% reduction (*n* = 327) in species richness (Fig. 2A). Significant decreases in species richness were observed for land-use change (24.8% decline, *n* = 61), species invasions (23.7% decline, *n* = 131), and habitat loss and fragmentation (14% decline, *n* = 60). Significant changes in species richness were not observed for nutrient addition (8.2% decline, *n* = 46) or temperature increase (3.6% decline, *n* = 28). Between-class heterogeneity was marginally insignificant (*Q*_b_ = 9.12, *P* = 0.058), suggesting that the magnitude of species loss slightly differs between the different disturbance type categories. When grouped according to experimental or observational study type, which only applied for species invasions and habitat loss, experimental studies had a slightly lower, yet not significantly different, fraction of decline in species richness than observational studies (Fig. 2B). This difference was more pronounced for species invasions, where experimental invasion studies had a lower decline in species richness losing an average of 11.2% less species (*n* = 16) than observational invasion studies, which lost an average of 24.2% of species (*n* = 116). In contrast, the fraction of decline in species richness between experimental and observational habitat loss studies was more similar, with experimental studies losing an average of 10.2% of species (*n* = 23) and observational studies losing an average of 17.1% of species (*n* = 37). The between-class heterogeneity was marginally insignificant (*Q*_b_ = 6.83, *P* = 0.078), suggesting that the fraction of decline in species richness slightly differs between the two study type categories.

**Figure 3:**
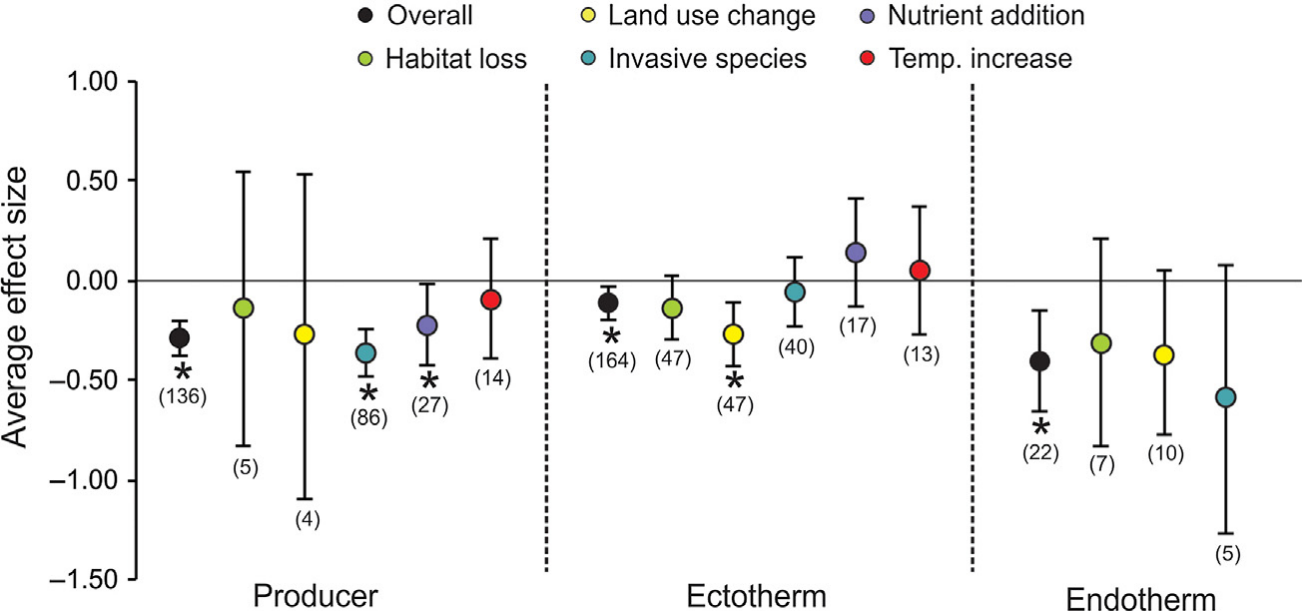
Change in species richness in species categories following anthropogenic disturbances. Average response ratios and 95% confidence intervals of species richness changes in producers, ectotherms, and endotherms across all disturbances and for each disturbance type. Values that significantly differ from zero, according to the 95% confidence intervals, are indicated with an asterisk. The values in parentheses represent the number of responses included in the analysis.

In general, the type of species affected by the disturbance influenced the fraction of change in species richness observed across all disturbances (*Q*_b_ = 10.59, *P* = 0.005), and when separated into the different disturbance categories (*Q*_b_ = 25.91, *P* = 0.011). Across all disturbances, endotherms showed a greater decline in species richness than ectotherms or producers (Fig. 3). Endotherms lost an average of 33.2% of species across all disturbances while ectotherms lost 10.5%, and producers lost 25.1%. While there was a significant decline in the species richness of endotherms across all disturbances, when the disturbances were separated, none showed a significant decline. The greatest decline in endotherm species was caused by species invasions (44.9%), followed by land-use change (30.5%) and habitat loss (36.7%).

Producer species richness only significantly declined from species invasions (30.3%) and nutrient addition (19.5%). Land-use change (22.2%), habitat loss (13%), and temperature increase (8.9%) all led to insignificant declines in producer species richness. In contrast, land-use change was the only disturbance to lead to significant decline in species richness in ectotherm species (24%). Habitat loss led to a slightly insignificant decline in ectotherm species (12.8%), while species invasions led to insignificant ectotherm species loss (5.2%), and nutrient addition and increases in temperature led to a small, yet insignificant, increase in ectotherm species richness (15.5% and 5.3%, respectively). Overall, species invasions was the only disturbance type to cause significantly different fractions of change in species richness between species categories resulting in a significantly greater decline in producer species richness (30.3%) compared with ectotherm species richness (5.2%).

**Figure 4:**
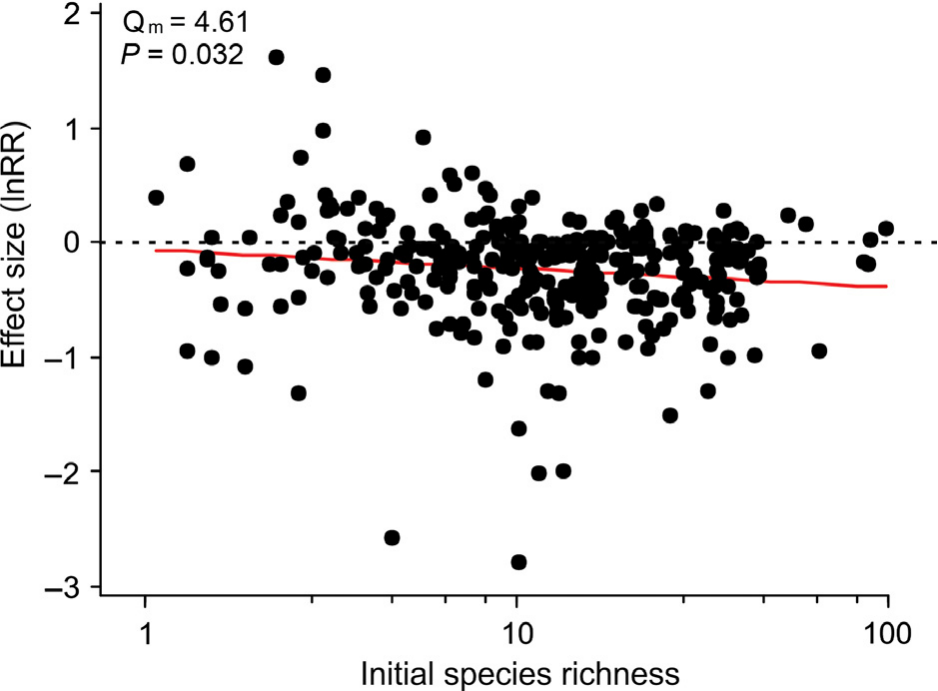
Relationship between initial species richness and change in species richness across all disturbances. Species richness in control is used as an indication of initial species richness. Model heterogeneity statistics (*Q*_m_) and corresponding *P*-values are shown.

Higher initial species richness was associated with greater species loss across all disturbances (*Q*_m_ = 4.61, *P* = 0.032; Fig. 4). At low initial richness values, disturbances were also generally associated with increases in species richness in disturbed habitats. When separated by disturbance type, there was no relation between change in species richness and initial species richness for any of the disturbances (Fig. S1).

There was no relationship between latitude and the fraction of change in species richness across all disturbances or for each disturbance category (Fig. S2). Experimental length also had no significant effect on the fraction of change in species richness (*Q*_m_ = 0.5, *P* = 0.48; Fig. S3). Heterogeneity statistics and corresponding *P*-values for all factors included in the meta-analysis are displayed in Table S1.

### Disturbances across biomes

**Figure 5:**
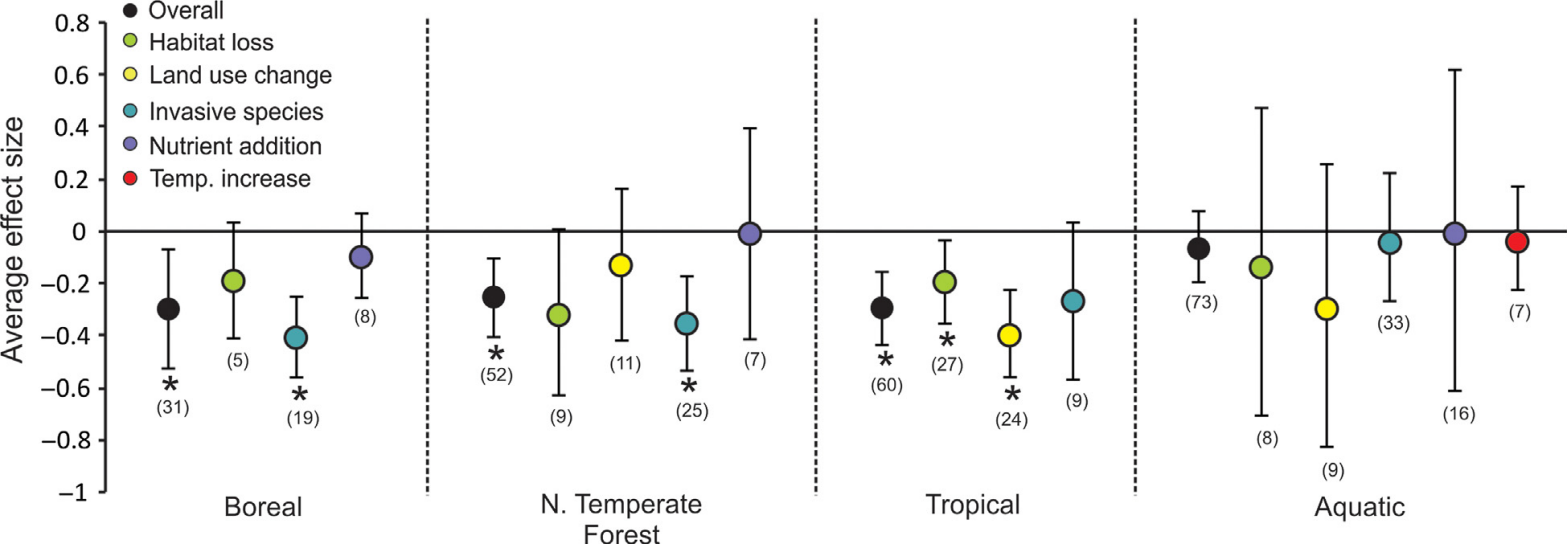
Change in species richness across Earth's biomes following anthropogenic disturbances. Response ratios of species richness across all disturbances and for each of the five disturbance types in boreal forest, northern temperate forest, tropical, and aquatic biomes. Values for each disturbance that significantly differ from zero according to the 95% confidence intervals are indicated with an asterisk. The values in parentheses represent the number of responses included in the analysis.

Our results also show that the vulnerability of an ecosystem's biodiversity differs across the Earth's biomes. Across all disturbances, significant decline in species richness was observed in all three of the terrestrial biomes we compared, and no significant change in species richness was observed in the aquatic biome (Fig. 5). This decline was greatest in the boreal forests with a 25.8% decline in species richness (*n* = 31), followed by the tropics (25.6% decline, *n* = 60), and northern temperate forests (22.5% decline, *n* = 52). Between-class heterogeneity was marginally significant (*Q*_b_ = 6.99, *P* = 0.072), suggesting that the fraction of decline in species richness slightly differs among the four biome categories across all disturbances.

None of the five disturbances led to significant change in species richness in the aquatic biome, and the effect of all disturbances did not differ from each other. Comparisons among the disturbance categories in the three terrestrial biomes revealed that the disturbances led to different fractions of change in species richness among the different biomes. Habitat loss led to significant declines in species richness in the tropics (25.6% decline, *n* = 27), yet did not lead to significant declines in either the boreal (17.2% decline, *n* = 17.22) or northern temperate forest (26.7%, *n* = 9) biomes. Land-use change was the disturbance that led to the greatest fraction of decline in species richness in the tropics (32.4% decline, *n* = 24), yet did not lead to significant decline in the northern temperate forest biome. Species invasions led to the greatest fraction of decline in species richness in both the boreal (33.5% decline, *n* = 19) and northern temperate forest (30% decline, *n* = 25) biomes, yet did not lead to significant decline in the tropics (23.7% decline, *n* = 9). Nutrient addition led to insignificant declines in species richness in both the boreal and northern temperate forest biomes.

### Publication bias

**Figure 6:**
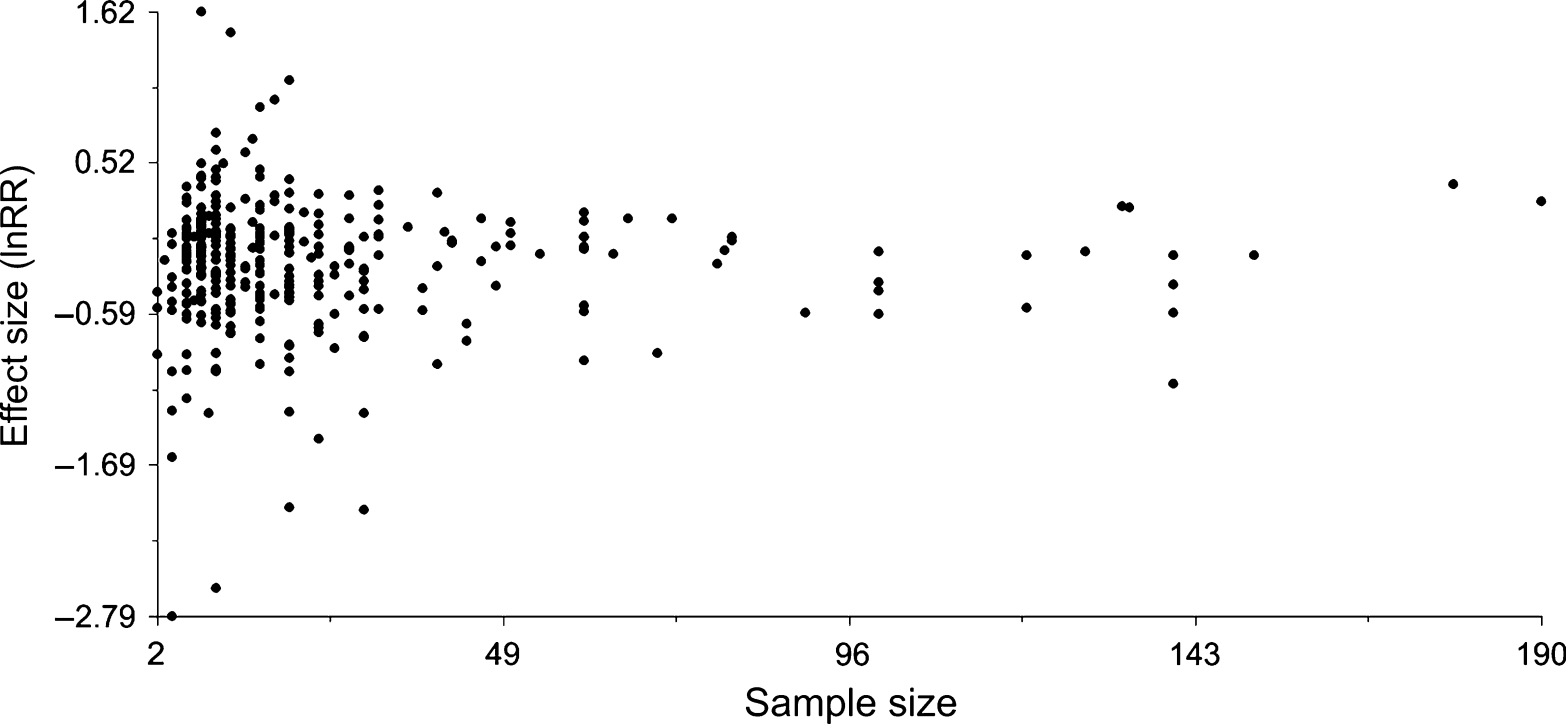
Funnel Plot used to determine the potential for publication bias. The effect sizes plotted against the corresponding sample sizes for each response in the dataset to identify asymmetry in the distribution of responses. Funnel shape suggests low potential for publication bias.

The funnel plot of sample size and effect size displays a clear funnel shape with a much greater spread of studies with small sample sizes and a decrease in this spread as sample size increases (Fig. 6). This funnel shape is what is expected if the studies are compiled from a random sampling with similar research methods (Moller and Jennions 2001), as it is expected that studies with smaller sample size will be less precise than those with large sample size. The clear funnel shape we see in this plot suggests that our dataset is unlikely to suffer from publication bias. The fail-safe number calculated for our dataset (5548.3) also indicates low publication bias. This number is over three times larger than Rosenthal's (1991) suggested number (5*327 + 10 = 1645) thus indicating that the negative effect of disturbance on species richness is very robust to publication bias.

## Discussion

### Fraction of change in species richness across disturbances

While habitat loss is widely cited as the leading cause of biodiversity decline (Vitousek et al. 1997; Pimm and Raven 2000; Millenium Ecosystem Assessment 2005) our results show that, at local scales, species invasions result in a fraction of change in species richness comparable to land-use change and greater than that caused by habitat loss/fragmentation. One potential explanation for this result lies in the difference in the fraction of change in species richness between observational and experimental studies of species invasions. Observational studies differ from experimental studies in many ways, one being the dispersal ability of species. Dispersal is likely limited in experimental plots while there is more environmental heterogeneity and dispersal potential in observational studies. A greater potential for replacement of lost individuals or species in observational studies implies that the fraction of decline in species richness might be lower. Our results reveal the opposite pattern with observational studies of species invasions resulting in a decline in species richness that was over two times greater than the decline observed in experimental invasion studies. This large disparity between study types did not occur for habitat loss. Observational disturbance studies are unable to completely control for multiple disturbances, and it is likely that the disturbed treatment will differ in other ways from the reference treatment. Therefore, our results suggest that observational studies of species invasions may be partially confounded by multiple disturbances. Invasive species often establish more frequently in disturbed rather than pristine habitats (Didham et al. 2005) and are often associated with other disturbances, such as nutrient addition (Kercher and Zedler 2004) or habitat disturbances (MacDougall and Turkington 2005). Thus, the high fraction of decline in species richness resulting from species invasions may in part be due to synergistic interactions with other disturbances (Brook et al. 2008). The large, negative effect that we found of land-use change on species richness is not surprising, as previous predictive studies have stressed the impact of land-use change, suggesting that it will be more significant than climate change, nitrogen deposition, and species invasions (Chapin et al. 2000; Sala et al. 2000).

### Change in species richness across taxa

Our analysis of the fraction of change in species richness between species categories shows that land-use change results in significant declines in species richness in ectotherms, marginally insignificant declines in species richness in endotherms, and insignificant declines in species richness in producers (Fig. 2). This result supports the hypothesis that disturbances that transform habitats, including land-use changes, habitat destruction, and habitat fragmentation are correlated with the extinctions of species in high trophic positions and with large body sizes (Holyoak 2000; Gonzalez et al. 2011).

Species invasions was the only disturbance that led to significant declines in species richness in producers, and this decline was greater than the decline of ectotherm species following species invasions (Fig. 3). Endotherm species loss following species invasions was greater than for both producers and ectotherms, yet the sample size was small (*n* = 5), compared with that of the producers (*n* = 86) and ectotherms (*n* = 40), and thus, the effect was not significant. These results suggest that species invasions are more likely to lead to extinctions of producer species than consumer species. A potential explanation of this strong effect of invaders on producer species relates to the nature of the invader species. The studies in our analysis that examined the effect of invasions on ectotherms and endotherms included those where an ectotherm or endotherm species was the invader as well as those where a producer species was the invader. Although not statistically significant, decline in ectotherm species richness was greater in studies where the non-native invader was a producer (5.8% decline, *n* = 27), compared with when an ectotherm species was the invader (0.5% increase, *n* = 13). This pattern was also seen in endotherms, with endotherm species experiencing a 47.8% decline in species richness (*n* = 3) following invasion by a producer species, and a 35% decline in species richness (*n* = 2) following invasion by an endotherm species. These results suggest that non-native species that impact the base of a food web have a stronger effect than higher trophic level invaders. Because all of the studies in our analysis that measured the effect of an invader on producers were those where the non-native invader was also a producer species, the strong effect of producer invaders was likely amplified due to the direct competition the non-native invader had with the native species for resources.

An important caveat to consider when examining changes in species richness between different studies is the difference in how finely resolved the taxonomic groups are. There is typically much better characterization among larger animals, such as mammals, compared with small animals, such as invertebrates. Because smaller species may not be as finely resolved, the magnitude of change in species richness in these species may be potentially underestimated. Across all disturbances, our results show that the decline in endotherm species richness is greater (33.2%) than the decline in ectotherm species richness (10.5%). While this could be due to the hypothesis that extinctions are more highly correlated with large bodied and high trophic level species (Holyoak 2000; Gonzalez et al. 2011), it could also be a result of a difference in how the studies included in our dataset characterized the species.

It is well established that diverse communities are generally more stable in terms of their biomass than communities with lower species richness (Tilman 1999; McCann 2000; Campbell et al. 2011). Our finding that higher initial species richness was associated with greater species loss suggests that the stabilizing role of high diversity on productivity (McCann 2000; Tilman et al. 2006) may not extend to biodiversity maintenance in the face of perturbations. That biodiversity is more difficult to maintain in diverse communities may be related to skewness of species-abundance distributions toward rare species in more diverse communities (Sankaran and McNaughton 1999). There is substantial evidence that rare species are more susceptible to extinction following a disturbance than common species (Davies et al. 2004; Lavergne et al. 2005; Gonzalez et al. 2011). Therefore, the high fraction of decline in species richness we found following habitat loss and species invasions may be, in part, due to the high richness of rare, extinction-prone species in these studies compared with the other disturbances.

### Change in species richness across biomes

We observed a similar fraction of decline in species richness across all disturbances in the three terrestrial biomes that we compared. However, while all terrestrial biomes experienced an overall significant decline in species richness, the aquatic biome experienced a much lower, and insignificant, decline across all disturbances. This suggests that the effect of anthropogenic disturbances on species richness is stronger in terrestrial ecosystems. The difference in food web structure and ecosystem properties between aquatic and terrestrial habitats suggests that these systems can differ in their response to disturbances. The very low effect of species invasions in the aquatic biome (2.4% decline) was surprising given the strong overall effect of invasions across all biomes (23.7% decline) and within each of the terrestrial biomes (boreal = 33.5%, northern temperate forest = 30%, and tropical = 23.7%). A potential explanation for this small effect of species invasions in the aquatic biome is that there may be facilitative interactions occurring between the invaders and native species. There is evidence that non-native species can facilitate native species and potentially lead to increases in native species richness (Simberloff and Von Holle 1999; Rodriguez 2006). The most common mechanism of non-native facilitation of native species is habitat modification, where the invader modifies the natural habitat to create new physical structures, which can benefit native species (Rodriguez 2006). One of the most familiar examples of habitat modification by an invader is the dense, complex colonies formed by invasive bivalves in aquatic ecosystems. These colonies have been shown to cause a shift from planktonic to benthic food webs (Simberloff and Von Holle 1999) and lead to increases in invertebrate diversity (Stewart and Haynes 1994). Of the 33 aquatic species invasion responses in our dataset, we found that the non-native invaders had a positive interaction with the native species in almost half of the responses (19 negative effects vs. 14 positive effects). While facilitative interactions between invaders and native species has been shown to occur almost equally in terrestrial and aquatic habitats (Rodriguez 2006), we did not find the same strong dichotomy in the direction of the effect of species invasions in the terrestrial responses from our dataset (82 negative effects vs. 15 positive effects). Therefore, our analysis suggests that positive interactions between invaders and native species may be more common in aquatic ecosystems.

While the fraction of decline in species richness across all disturbances was similar among the three terrestrial biomes, we found variation among the biomes in terms of the disturbances that had the largest impact on species richness (Fig. 5), suggesting that the effects of human-caused disturbances are not uniform across the Earth's biomes. The decline in species richness caused by both land-use change and habitat loss was only significant in the tropical biome. This may be due to the extremely high level of taxonomic diversity in tropical biomes (Myers et al. 2000), which is particularly affected by a reduction in available living space. On the other hand, species invasions were the only disturbance to lead to significant decline in species richness in the northern temperate forest and boreal forest biomes. This suggests that species in these biomes are more robust to reduced habitat area, but may be vulnerable to competition for resources imposed by invaders.

Previous attempts to estimate and predict the magnitude of species loss resulting from different human-caused disturbances have relied heavily on expert opinion (e.g., Sala et al. 2000). In contrast, the estimates of declines in species richness presented here are based on empirical studies. In Sala et al. (2000), the authors predict future biodiversity change for five drivers of biodiversity decline (land use, atmospheric CO_2,_ nitrogen deposition, climate, and biotic exchange) in 11 terrestrial biomes along with lakes and streams. To make these predictions, they combine the expected changes in the five drivers with the expected impact of each driver on biodiversity loss. Sala et al. (2000) uses knowledge from experts to estimate the biodiversity impact of each driver in each biome, ranking the estimates from a low impact on biodiversity (1) to a high impact on biodiversity (5). While studies such as Sala et al. (2000) and the present meta-analysis differ in many respects including spatial scale and as such are not directly comparable, a number of the similarities and differences in the results of the two studies are interesting. While land-use change is estimated in Sala et al. (2000) to lead to more species loss across all biomes than any other disturbance, we only find significant declines in species richness resulting from land-use change in the tropics. Species invasions show a much stronger effect on species richness in northern temperate forests and boreal forest biomes based on the meta-analysis presented here than land-use change or habitat loss. Additionally, Sala et al. (2000) predict a relatively low impact of species invasions in these biomes. Our results based on empirical values of change in species richness show that the effect of species invasions on species richness will be much greater than is currently estimated by expert knowledge and that the effects of species invasions may be comparable to those of land-use change and habitat loss. While it is evident from our analysis that human-caused disturbances do not all contribute to the same fraction of decline in species richness in each biome, the large effect of species invasions stresses the significant impact that non-native species have on ecosystems.

An important caveat to consider when comparing our empirical estimates of change in species richness to estimates of global biodiversity change, such as those made by Sala et al. (2000) is how differences in spatial scale can impact the patterns of biodiversity loss. A variety of species richness patterns have been shown to be dependent upon spatial scale. These include differences in the strength or shape of the relationship between diversity and productivity (Chase and Leibold 2002), diversity and latitude (Hillebrand 2004), and diversity and altitude (Rahbek 2005) between local and regional scales. With spatial scale playing a large role in the strength of several species richness relationships, the effects of anthropogenic disturbances on the magnitude of species loss may also be scale-dependent, and thus the strength of the effects we found may differ at the global scale. It is possible that a disturbance might decrease local species richness, but increase regional species richness, as could be the case for the effects of nutrient addition if the scale-dependent diversity–productivity relationship holds true (Chase and Leibold 2002). A further understanding of the scale dependence of anthropogenic disturbances on the magnitude of species loss will be essential in order to make future biodiversity loss predictions at the global scale.

The latitudinal gradient in species richness from the polar to equatorial regions has been demonstrated for a wide variety of species and is one of the most fundamental patterns of biodiversity (Rosenzweig 1995; Willig et al. 2003). It has been suggested that biodiversity is potentially more difficult to maintain in diverse communities, due to these communities containing many rare species that are more susceptible to extinction following a disturbance (Sankaran and McNaughton 1999; Davies et al. 2004). Therefore, we would expect to find a latitudinal gradient in the fraction of change in species richness following disturbances, with low latitude regions that contain greater biodiversity experiencing a greater decline in species richness. However, we did not observe latitudinal gradients in the fraction of change in species richness for any of the five disturbances (Fig. S2). This suggests that while low latitude regions may be more susceptible to species loss due to their high biodiversity, the relative fraction of species richness decline does not differ from higher latitude regions with lower diversity. The issue of spatial scale may also be playing a role in the absence of a latitudinal gradient in our results. As discussed above, the latitudinal diversity gradient is known to differ between spatial scales, with a stronger and steeper relationship at the regional scale compared with the local scale (Hillebrand 2004). Because our meta-analysis examined change in species richness at the local scale, it is possible that a similar relationship exists, with a weaker relationship between latitude and the magnitude of species loss following anthropogenic disturbances at the local scale compared with what we might observe at a larger, regional scale.

### Knowledge gaps

In compiling the dataset of disturbance studies for this meta-analysis, we found major data gaps, making it impossible to make comparisons of the effects of disturbance types across all of Earth's biomes. These gaps are the result of research intensity skewed toward different disturbances for different biomes, rather than research aimed at gaining a broad understanding of global effects of disturbance. While disturbance-mediated biodiversity loss has been well studied in some biomes, for example boreal and northern temperate forests, information is largely lacking for disturbances in others. For example, climate change is extensively studied in the arctic and alpine biomes yet few studies have addressed the effects of increases in temperature on biodiversity in northern temperate forest or tropical biomes. Likewise, while species invasions have been well studied in many of Earth's biomes, data are lacking for arctic and alpine biomes. These shortcomings limit our ability to compare the major drivers of biodiversity loss across the Earth's biomes and need to be addressed in order to accurately assess how anthropogenic disturbances affect biodiversity at the global scale.

These knowledge gaps seriously hinder our ability to make accurate predictions of future biodiversity change. These shortcomings should be considered when using empirical values of species loss to make predictions of biodiversity change. It will be necessary for future studies to focus on exploring biodiversity changes in the areas where knowledge gaps exist to further improve these projections of future biodiversity change.

### Future directions

In this study, we used species richness to measure the magnitude of biodiversity change. Species richness is the most common biodiversity measure used in disturbance studies, and while it provides a measure of the magnitude of species loss, it is unable to account for the complex changes in composition and community structure that can take place following disturbances (Mendenhall et al. 2012). For example, following deforestation in Costa Rica for agricultural activity bird species richness did not significantly differ between forested and agricultural habitats, suggesting that the deforestation did not have the large negative impact on the community that would be anticipated (Daily et al. 2001). However, community composition differed greatly between habitats, with the natural forest and agricultural area showing two distinct communities (Mendenhall et al. 2011). Changes in the abundance distributions of species in disturbed ecosystems are also important indicators of change. Another overlooked problem when using only average values of change in species richness as a metric of biodiversity is that disturbances can also affect the consistency, or predictability, of a response (Fraterrigo and Rusak 2008; Murphy and Romanuk 2012). Response predictability is a relatively unexplored consequence of disturbances but an understanding of response predictability changes can help to better interpret the ecological effects of disturbances (Murphy and Romanuk 2012). Future disturbance studies should concentrate on including alternative measures of biodiversity, such as community composition, along with species richness to obtain a clearer understanding of how different types of human-caused disturbances affect biodiversity.

## Supporting Information

**Figure S1.** Relationship between initial species richness and the change in species richness for habitat loss (A), land-use change (B), species invasion (C), nutrient addition (D), and temperature increase (E).

**Figure S2.** Relationship between latitude and the change in species richness following anthropogenic disturbances.

**FigureS3.** Relationship between experimental length (days) and change in species richness for experimental studies.

**Table S1.** Heterogeneity statistics and corresponding *P*-values for each of the categorical and continuous factors included the meta-analysis.

**Data S1.** Dryad data.
