# Supplementary figures and images for: A meta-analysis of declines in local species richness from human disturbances
Source: Ecol Evol. 2013 Dec 12;4(1):91–103. doi: 10.1002/ece3.909 (PMC3894891; doi:10.1002/ece3.909)

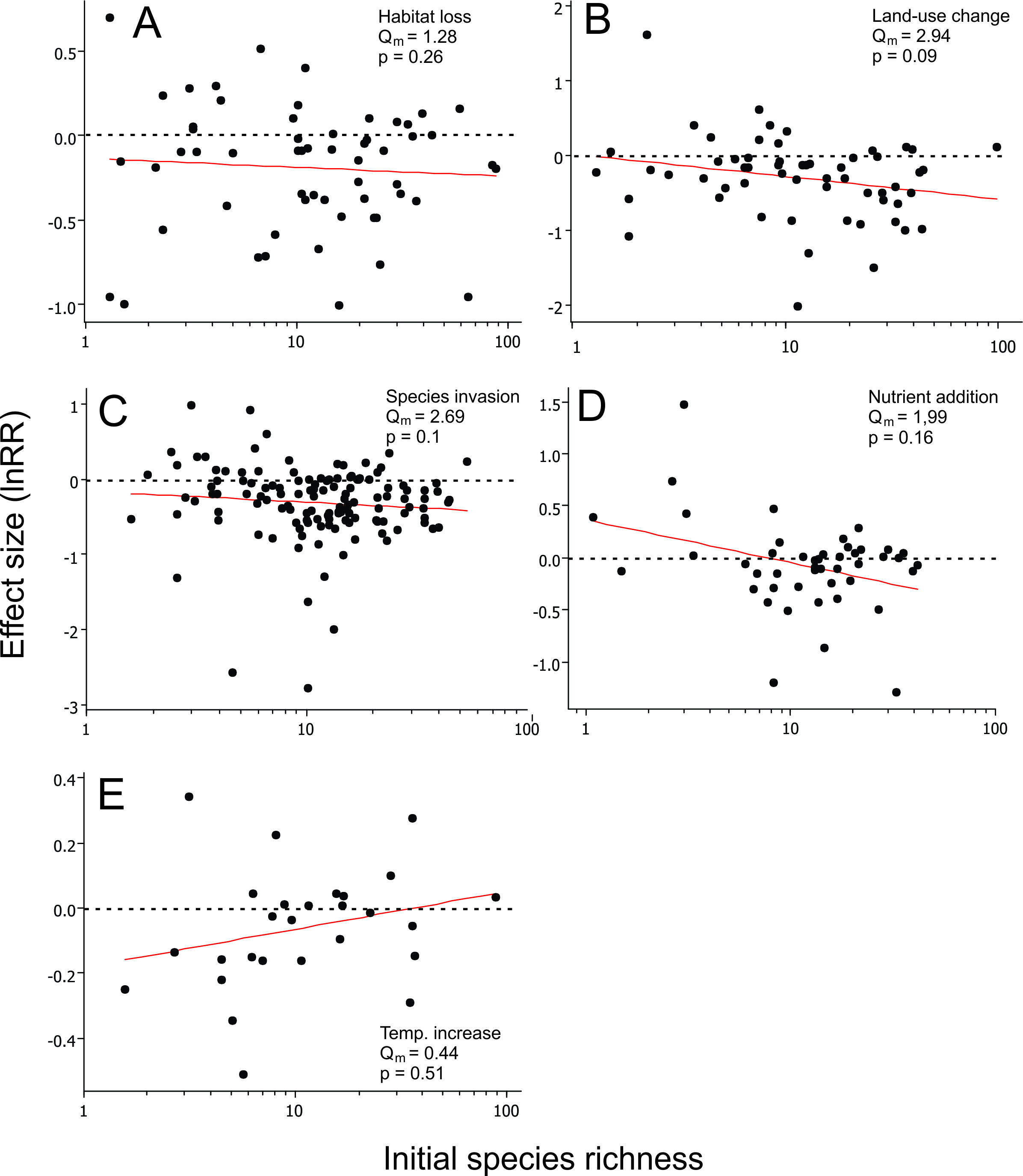

Supplement: Supplementary file 1 [file ece30004-0091-SD1.jpg]

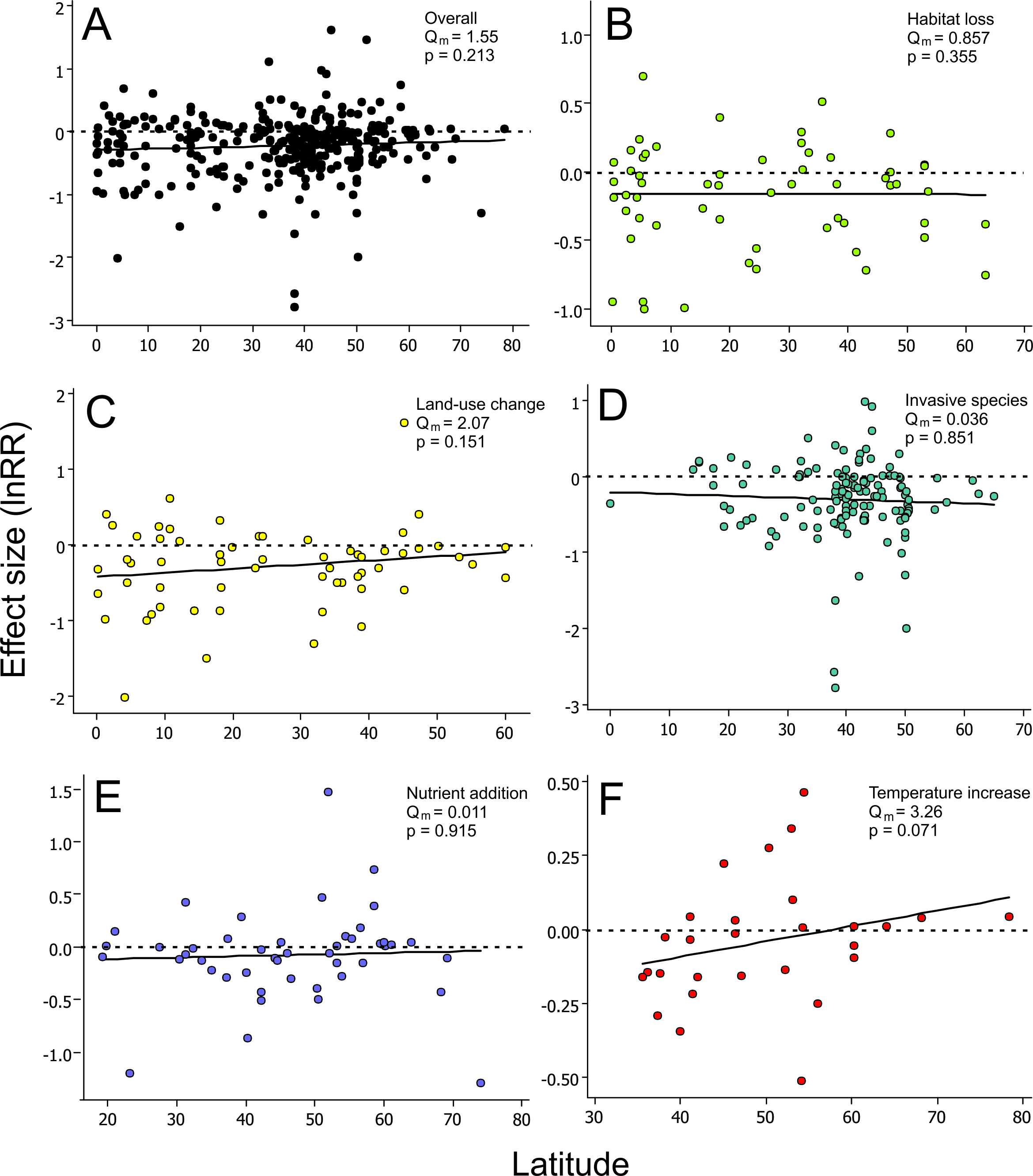

Supplement: Supplementary file 2 [file ece30004-0091-SD2.jpg]

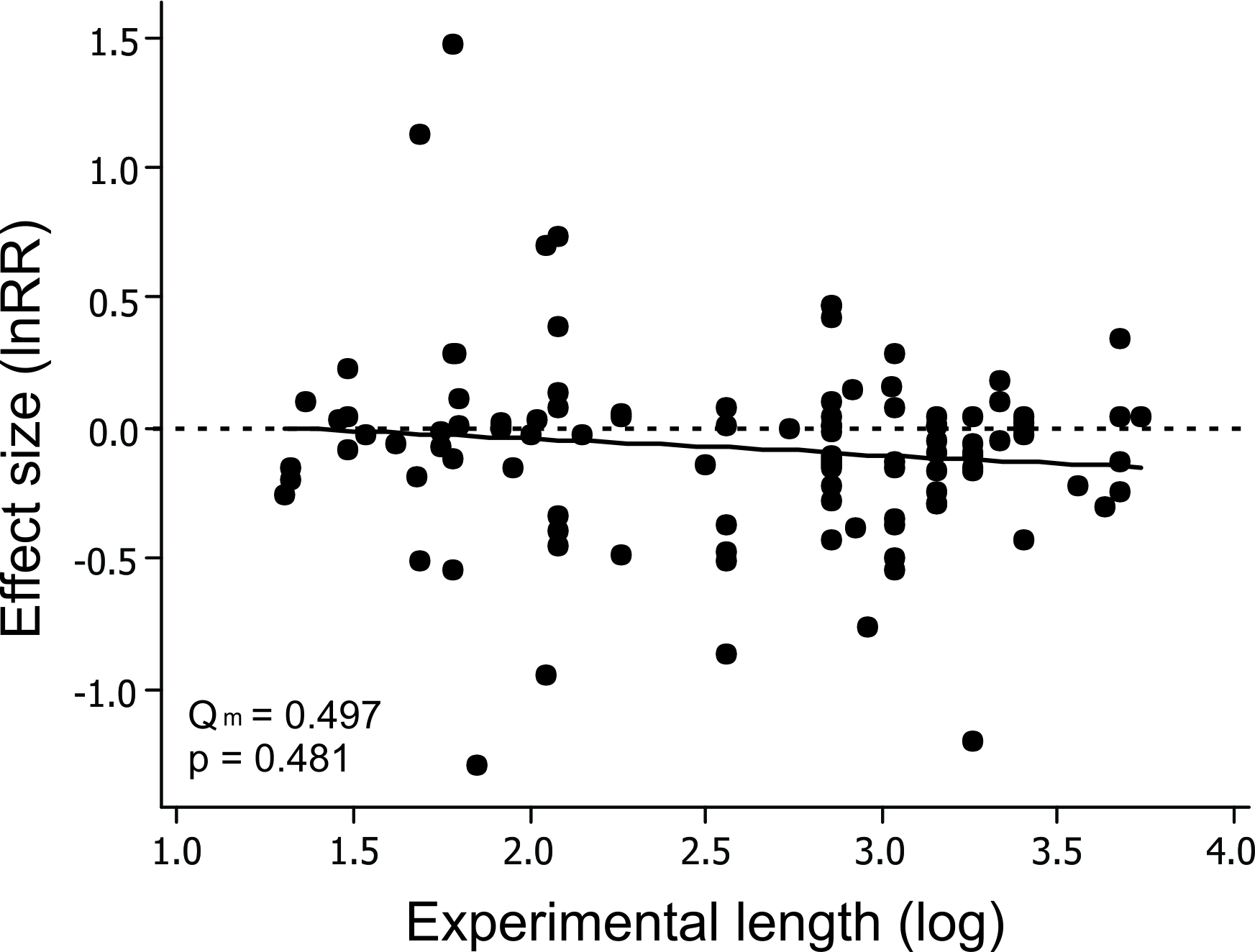

Supplement: Supplementary file 3 [file ece30004-0091-SD3.jpg]
