# Supplementary material for: A meta-analysis of declines in local species richness from human disturbances
Source: Ecol Evol. 2013 Dec 12;4(1):91–103. doi: 10.1002/ece3.909 (PMC3894891; doi:10.1002/ece3.909)
Supplement: Supplementary file 4 [file ece30004-0091-SD4.docx]

**Table S1:**

**Table S1.** Heterogeneity statistics and corresponding p-values for each of the categorical and continuous factors included the meta-analysis.

|  |  |  |  |  |  |  | | |  |
| --- | --- | --- | --- | --- | --- | --- | --- | --- | --- |
| Categorical factors  factors ana |  | **Q_t_** | **p** | **Q_b_** | **p** | **Q_w_** | **p** | | |
|  | Disturbance type | 219.10 | 1.00 | 9.12 | 0.058 | 209.98 | 1.00 | | |
|  | Study type | 249.48 | 0.002 | 6.83 | 0.078 | 242.66 | 0.004 | | |
|  | Trophic category | 209.52 | 1.00 | 10.59 | 0.005 | 198.92 | 1.00 | | |
|  | Trophic x Disturbance | 214.82 | 1.00 | 25.91 | 0.011 | 188.90 | 1.00 | |  |
|  | Biome type | 157.40 | 0.99 | 6.99 | 0.072 | 150.39 | 0.99 | |  |
|  | Biome x Disturbance | 162.32 | 0.99 | 12.88 | 0.302 | 149.45 | 0.99 | |  |
| Initial SR (continuous) |  | **Q_t_** | **p** | **Q_m_** | **p** | **Q_e_** | **p** | |  |
|  | All disturbances | 200.59 | 1.0 | 4.61 | 0.032 | 195.98 | 1.00 | |  |
|  | Habitat loss | 71.07 | 0.099 | 1.282 | 0.257 | 69.79 | 0.102 | |  |
|  | Land-use change | 77.10 | 0.039 | 2.94 | 0.086 | 74.16 | 0.053 | |  |
|  | Species invasion | 158.23 | 0.041 | 2.69 | 0.101 | 155.54 | 0.049 | |  |
|  | Nutrient addition | 16.72 | 0.99 | 1.99 | 0.159 | 14.72 | 0.999 | |  |
|  | Temp. increase | 26.29 | 0.447 | 0.441 | 0.506 | 25.85 | 0.416 | |  |
| Latitude (continuous) |  | **Q_t_** | **p** | **Q_m_** | **p** | **Q_e_** | | **p** |  |
|  | All disturbances | 277.06 | 0.98 | 1.55 | 0.21 | 275.51 | | 0.98 |  |
|  | Habitat loss | 69.59 | 0.14 | 0.86 | 0.35 | 68.74 | | 0.14 |  |
|  | Land-use change | 91.93 | 0.006 | 2.06 | 0.15 | 89.86 | | 0.008 |  |
|  | Species invasion | 153.11 | 0.09 | 0.036 | 0.85 | 153.12 | | 0.08 |  |
|  | Nutrient addition | 39.21 | 0.71 | 0.01 | 0.92 | 39.2 | | 0.68 |  |
|  | Temp. increase | 29.15 | 0.35 | 3.26 | 0.07 | 25.89 | | 0.47 |  |

| Exp. length (continuous) |  | **Q_t_** | **p** | **Q_m_** | **p** | **Q_e_** | **p** |
| --- | --- | --- | --- | --- | --- | --- | --- |
|  | All disturbances | 36.69 | 1.00 | 0.497 | 0.481 | 36.19 | 1.00 |
