# Supplementary material for: A meta-analysis of declines in local species richness from human disturbances
Source: Ecol Evol. 2013 Dec 12;4(1):91–103. doi: 10.1002/ece3.909 (PMC3894891; doi:10.1002/ece3.909)
Supplement: Supplementary file 6 [file ece30004-0091-SD6.docx]

Data S1. Dryad data.
